# Supplementary material for: Assortative mixing in micro-architecturally annotated brain connectomes
Source: Nat Commun. 2023 May 18;14:2850. doi: 10.1038/s41467-023-38585-4 (PMC10195875; doi:10.1038/s41467-023-38585-4)
Supplement: Supplementary file 1 — Supplementary Information [file 41467_2023_38585_MOESM1_ESM.pdf]

Annotations		z-assortativity	p_{spin}
5HTT	5HT2a	-4.68	0.0001
5HTT	5HT4	-4.23	0.0001
5HTT	CB1	-3.16	0.049
5HTT	MU	-3.67	0.012
NAT	MU	-3.28	0.0001
NAT	5HT2a	-7.81	0.0001
NAT	5HT4	-5.84	0.0001
NAT	M1	-4.25	0.020
NAT	mGluR5	-3.54	0.044
VAChT	5HT2a	-4.63	0.0001

TABLE S1. **Significantly disassortative mixing between receptors and transporters in FC** | Significance was evaluated using a permutation test with spatial autocorrelation-preserving null annotations ($p_{\text{spin}} < 0.05$, two-sided, FDR-corrected).

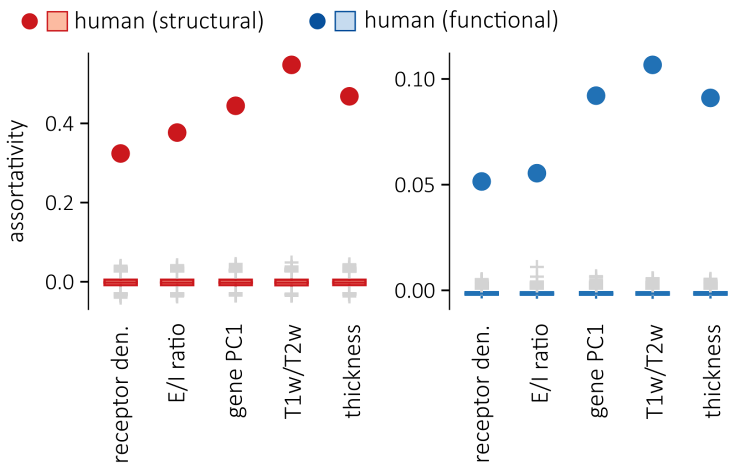

Figure S1. **Assortativity relative to spatially-naive nulls** | Assortativity scores of empirical annotations (points) compared to the assortativity scores of $n = 10,000$ null annotations generated via spatially-naive permutation for the structural (blue) and functional (red) connectomes. As a result of the brain's spatial embedding, the p-values obtained when comparing the empirical assortativity scores against those obtained using these spatially-naive null models are heavily inflated ($p = 0.0001$, two-sided, FDR-corrected).

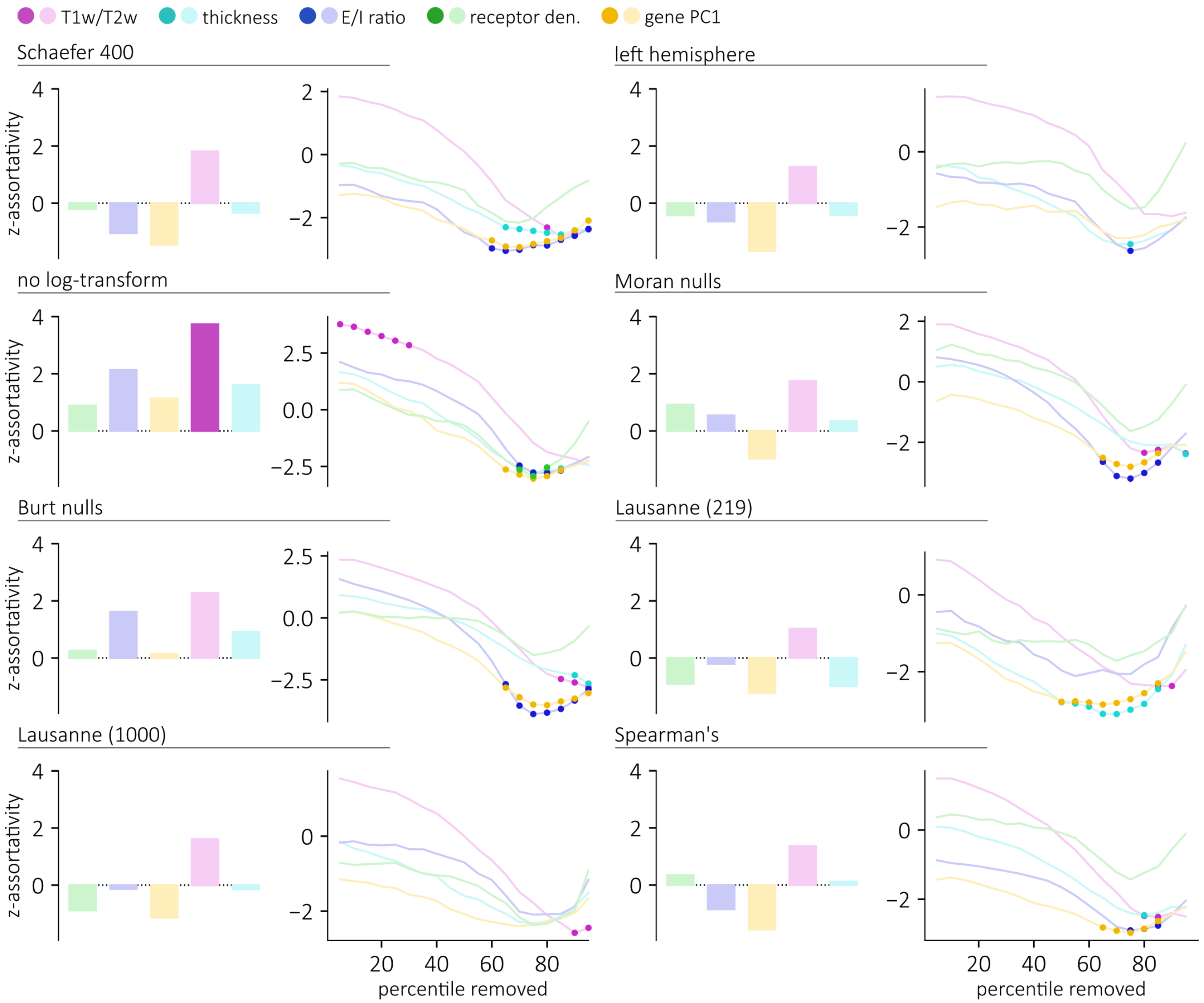

Figure S2. **Sensitivity and replication (homophilic mixing) |** To ensure that the results obtained in the structural connectome are not sensitive to our processing choices, we replicated our experiments using the 400 nodes Schaefer parcellation [89], using the left-hemisphere connectome, using edge weights that were not log-transformed, using Moran nulls and Burt nulls instead of the spin nulls and using an additional independently-acquired dataset (Lausanne), parcellated at a low- (219 nodes) and high-resolution (1000 nodes) according to the Cammoun parcellation [96]. Furthermore, to explore whether non-linear relationships exists between the annotations of connected nodes, we replicated the results using a rank-based measure of assortativity (Spearman's ρ). For each sensitivity and replication experiment, and for each annotation, we re-computed the standardized assortativity scores (left). Dark-colored bars denote z-assortativity scores that are statistically significant, relative to distributions of $n = 10,000$ spatial autocorrelation-preserving null annotations ($p < 0.05$, two-sided, FDR-corrected). We also re-computed the standardized assortativity scores across thresholded connectomes where a given percentile of the shortest connections are removed (right). Dark-colored circles indicate percentiles at which z-assortativity scores are statistically significant, again relative to a distribution of $n = 10,000$ spatial autocorrelation-preserving null annotations ($p < 0.05$, two-sided, FDR-corrected).

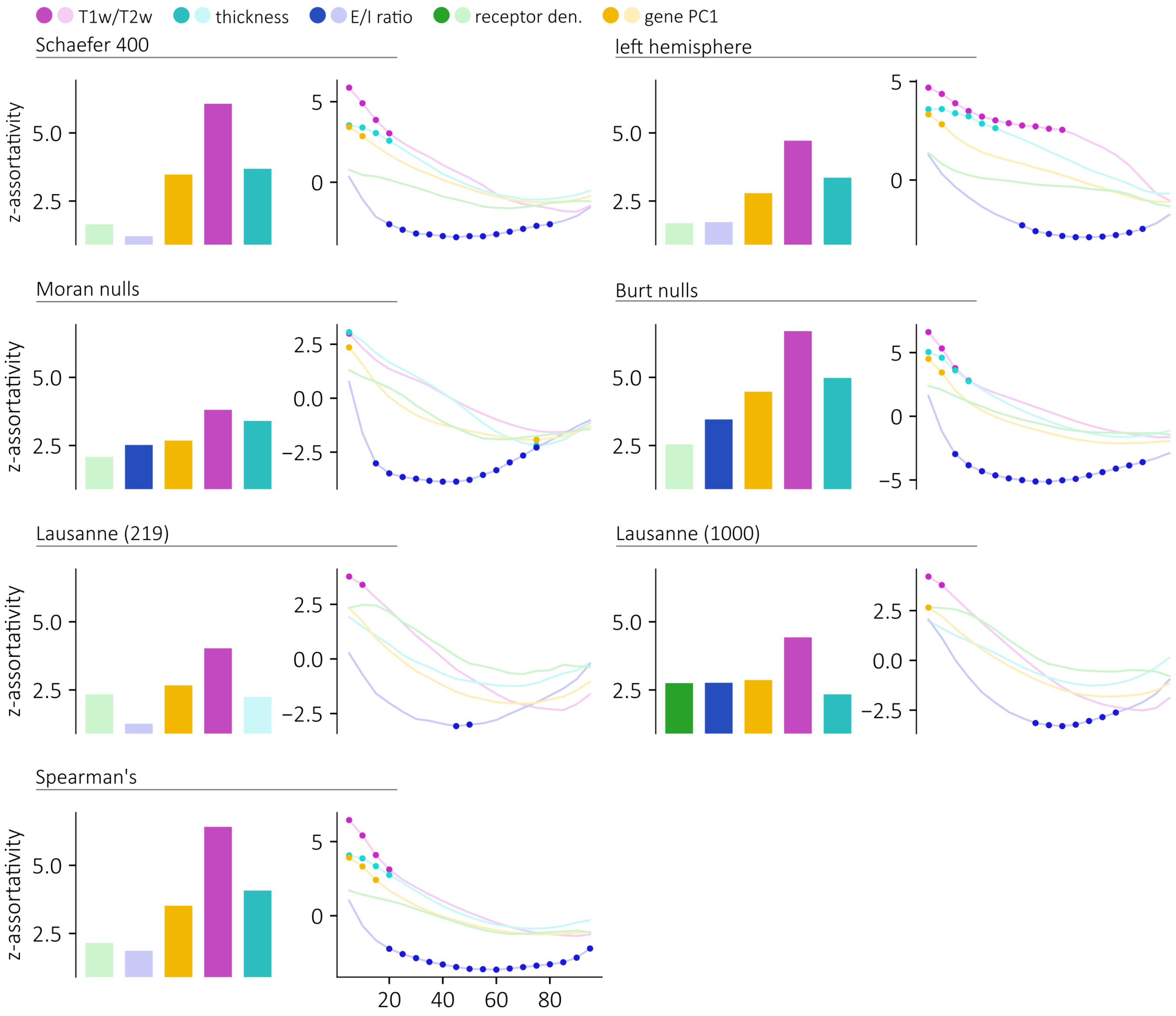

Figure S3. **Sensitivity and replication (homophilic mixing) |** To ensure that the results obtained in the functional connectome are not sensitive to our processing choices, we replicated our experiments using the 400 nodes Schaefer parcellation [89], using the left-hemisphere connectome, using Moran nulls and Burt nulls instead of the spin nulls, and using an additional independently-acquired dataset (Lausanne), parcellated at a low- (219 nodes) and high-resolution (1000 nodes) according to the Cammoun parcellation [96]. Furthermore, to explore whether non-linear relationships exists between the annotations of connected nodes, we replicated the results using a rank-based measure of assortativity (Spearman's ρ). For each sensitivity and replication experiment, and for each annotation, we re-computed the standardized assortativity scores (left). Dark-colored bars denote z-assortativity scores that are statistically significant, relative to distributions of $n = 10,000$ spatial autocorrelation-preserving null annotations ($p < 0.05$, two-sided, FDR-corrected). We also re-computed the standardized assortativity across thresholded connectomes where a given percentile of the shortest connections are removed (right). Dark-colored circles indicate percentiles at which z-assortativity scores are statistically significant, again relative to a distribution of $n = 10,000$ spatial autocorrelation-preserving null annotations ($p < 0.05$, two-sided, FDR-corrected).

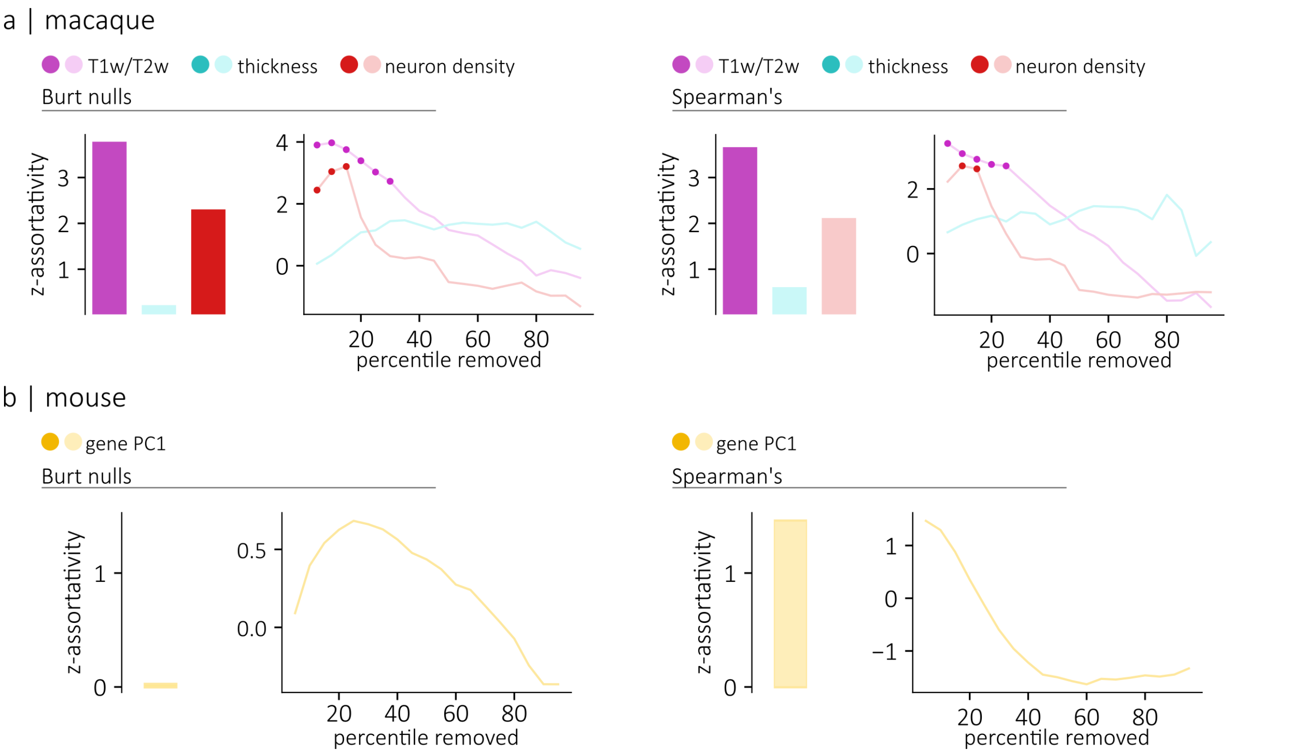

Figure S4. **Sensitivity and replication (homophilic mixing) |** To ensure that the results obtained in the macaque (**a**) and in the mouse (**b**) connectomes are not sensitive to the spatial autocorrelation-preserving null model used, we replicated our experiments using Burt nulls. Furthermore, to assess whether, more generally, there exists monotonic relationships between the annotations of connected nodes, we computed the rank-based assortativity coefficient (Spearman's ρ) of each annotation. For each sensitivity and replication experiment, and for each annotation, we re-computed the standardized assortativity scores (left). Dark-colored bars denote z-assortativity scores that are statistically significant, relative to distributions of $n = 10,000$ spatial autocorrelation-preserving null annotations ($p < 0.05$, two-sided, FDR-corrected). We also re-computed the standardized assortativity across thresholded connectomes where a given percentile of the shortest connections are removed (right). Dark-colored circles indicate percentiles at which z-assortativity scores are statistically significant, again relative to a distribution of $n = 10,000$ spatial autocorrelation-preserving null annotations ($p < 0.05$, two-sided, FDR-corrected).

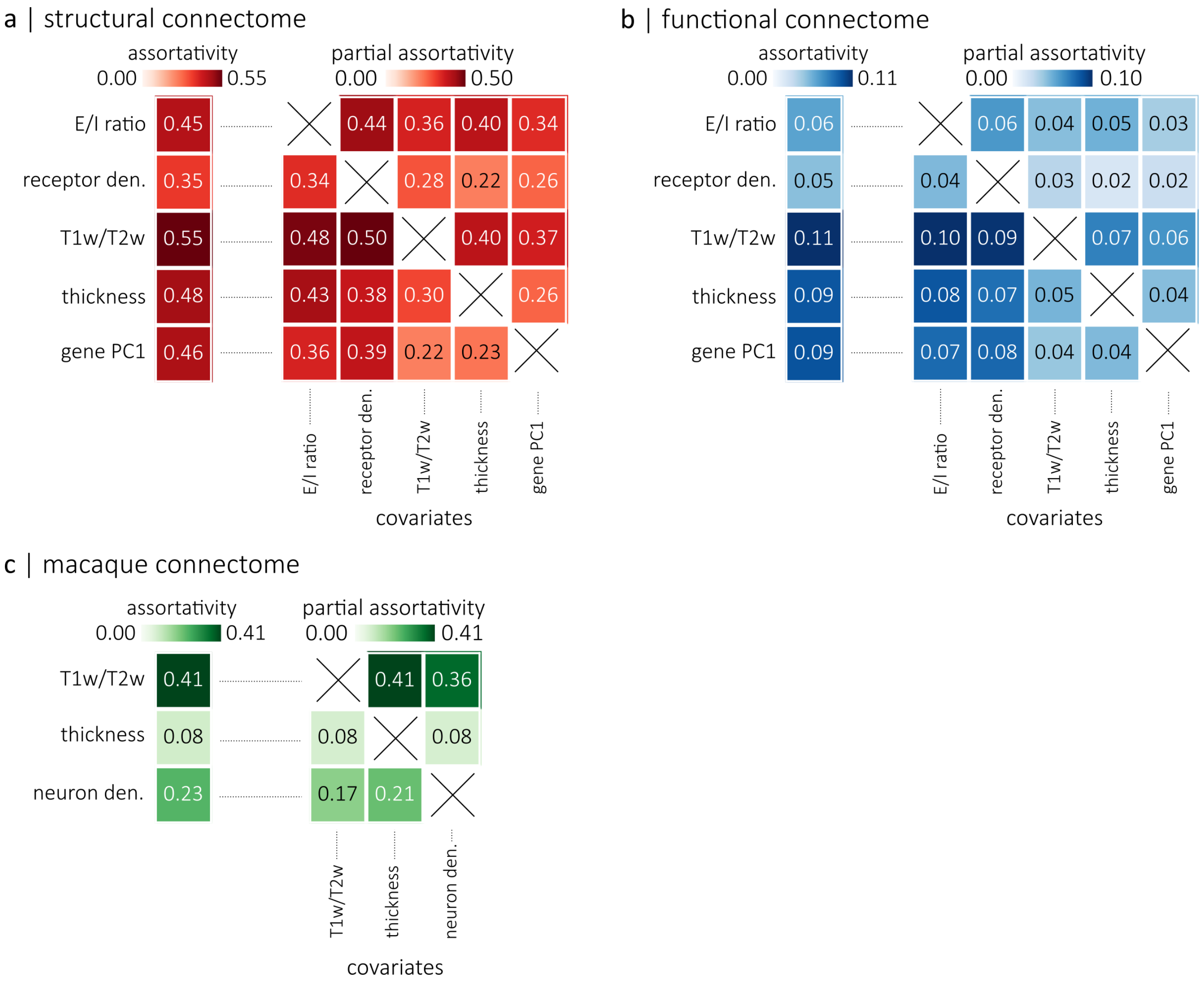

Figure S5. **Partial assortativity |** To explore whether the assortativity scores obtained for specific annotations are confounded by other annotations, we computed the partial assortativity of all annotations. Namely, for each annotation, we regressed out the potential contributions of the other annotations and computed a weighted correlation between the residuals. Partial assortativity scores are shown for the annotations of the human structural **(a)**, human functional **(b)** and macaque **(c)** connectomes. The left-most columns show the assortativity results presented in the main text. The heatmaps on the right show the partial assortativity scores. Each row consists of the partial assortativity scores of a given annotation, with the scores of the remaining annotations (covariates) regressed out. By computing the significance of the partial assortativity scores with a permutation test (two-sided, FDR-corrected), we find significant partial correlations for all annotations of the human connectomes ($p < 0.05$). In other words, we find that the assortativity scores obtained are not confounded by other annotations. For the macaque connectome, the partial assortativity scores obtained for cortical thickness were not significant, which is consistent which our main findings. However, the assortativity scores obtained for T1w/T2w ratio and neuron density were still significant after regressing out the other annotations ($p < 0.05$).

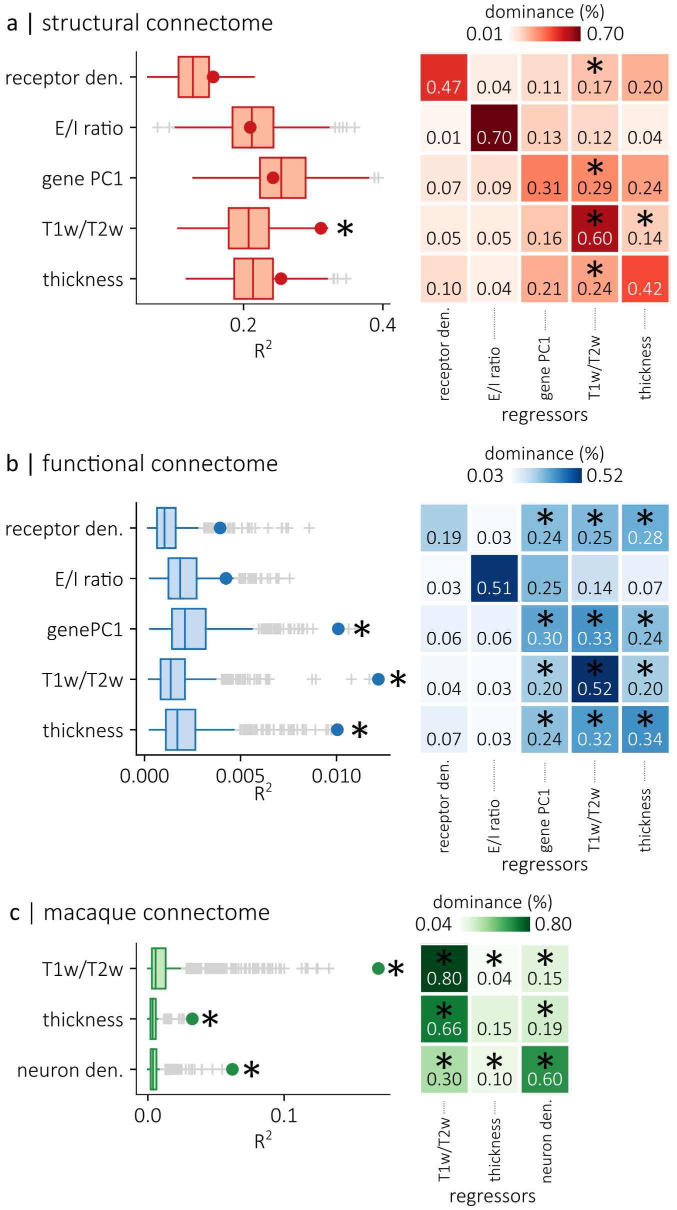

Figure S6. **Multiple linear regression and dominance analysis |** For each annotation in each network, we developed a regression model to predict the annotation score of a brain region from the annotation scores (across all attributes) of its connected neighbours. The coefficient of determination (R^2) was used to evaluate the fit of each model. R^2 values obtained with the empirical annotations (points) were compared to the R^2 values obtained using n=1,000 spatial autocorrelation-preserving null permutations (boxplots). Significant fits ($p < 0.05$, two-sided), evaluated using permutation tests and corrected for multiple comparisons (FDR correction), are denoted with asterisks. Permutations consisted in spin permutations for the human connectomes, and were generated from Moran nulls for the macaque connectome. To preserve the dependence between attributes, the same permutation was used across all dependent variables of each model. Dominance analysis was also used to evaluate the contribution of each regressor in each regression model. The significance of these contributions was again evaluated against spatial autocorrelation-preserving permutations. Asterisks denote significant contributions ($p < 0.05$, two-sided). **(a)** In the structural connectome, the regression model using empirical annotations was better at predicting T1w/T2w ratio than regression models built using null annotations ($R^2 = 0.31, p_{\text{spin}} = 0.046$). Dominance analysis highlights significant contributions from T1w/T2w ratio and cortical thickness. **(b)** In the functional connectome, our regression models were better at predicting T1w/T2w ratio ($R^2 = 0.012, p_{\text{spin}} = 0.001$), cortical thickness ($R^2 = 0.010, p_{\text{spin}} = 0.02$) and gene PC1 ($R^2 = 0.010, p_{\text{spin}} = 0.02$). Dominance analysis shows that these three annotations significantly contribute to the performance of each model **(c)**. For the macaque connectome, our regression models performed significantly better than those built using null annotations (T1w/T2w: $R^2 = 0.17, p_{\text{moran}} = 0.001$; thickness: $R^2 = 0.03, p_{\text{moran}} = 0.001$, neuron density: $R^2 = 0.06, p_{\text{moran}} = 0.001$). Boxplots represent the 1st, 2nd (median) and 3rd quartiles of the null distributions; whiskers represent the non-outlier endpoints of the distribution; and + symbols represent outliers.

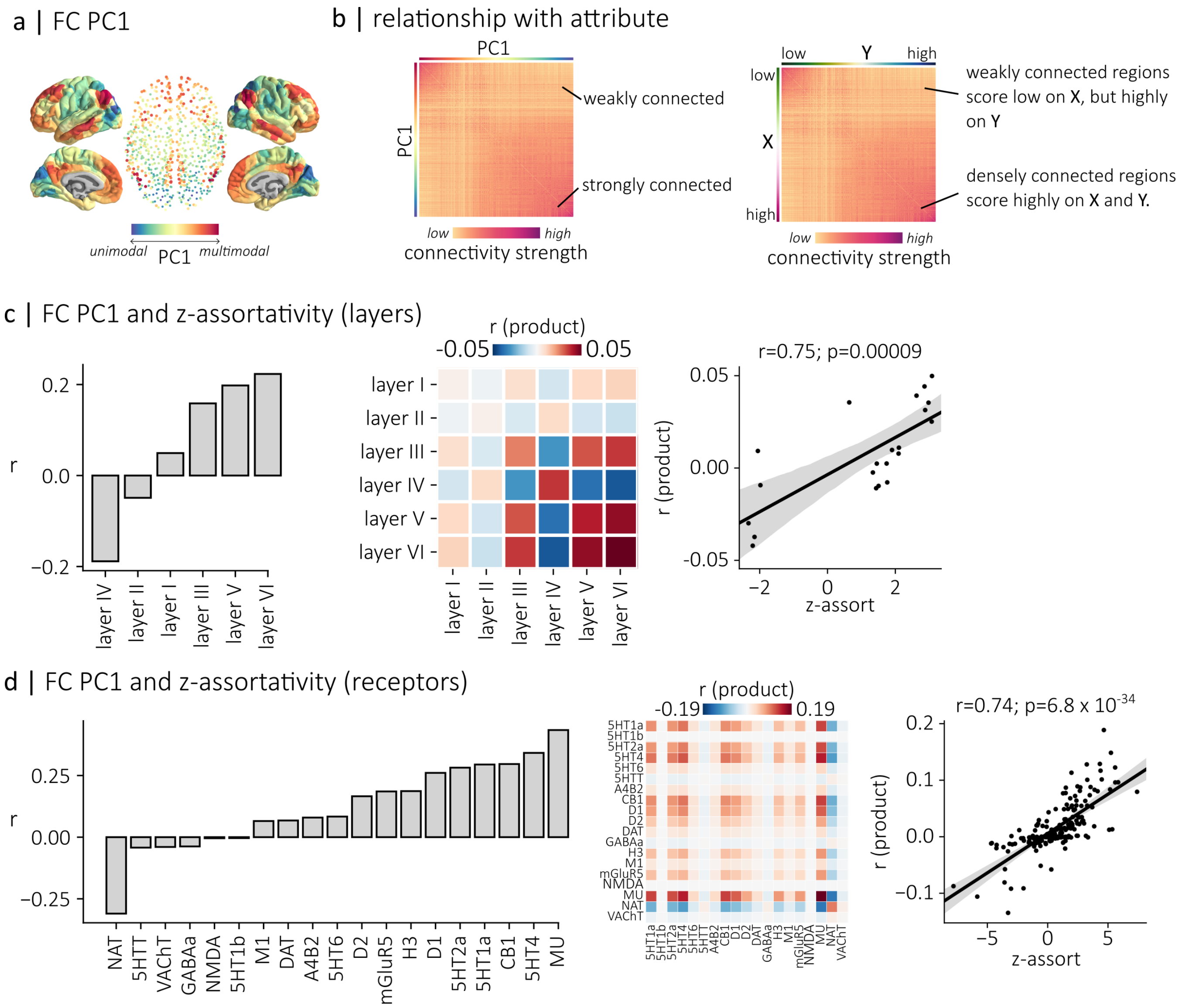

Figure S7. **Relationship between the principal axis of connectivity and assortativity | (a)** The principal axis of connectivity in the human functional connectome (FC PC1) is described as a functional hierarchy ranging from unimodal to transmodal cortex. It can be computed by applying principal component analysis on the functional connectivity matrix **(b)** Brain regions are ordered along FC PC1 based on their connectivity profile: densely interconnected regions are grouped together on this axis (left). When a pair of annotations is correlated with FC PC1 (right), then densely interconnected regions have similar annotation scores (e.g. score highly on two annotations X and Y). As a result, this pair of annotations will be assortative on the functional connectome. **(c)** We computed the Pearson correlation between the laminar thickness brain maps and FC PC1 (left). Layers II and IV are positively correlated with FC PC1 while layers VI, V, III and I are negatively correlated with FC PC1. For each pair of laminar thickness maps, we then evaluated whether they had similar or opposing relationships with FC PC1 by computing the product of their correlations with FC PC1 (middle). The products were then compared to the z-assortativity of each pair of annotations (right). We find a significant relationship between the two ($r(19) = 0.75$, $p = 0.00009$, CI=[0.47, 0.89], two-sided). In other words, we show that the assortativity between pairs of laminar thickness maps can be explained by their relationship with the functional hierarchy. **(d)** We also evaluated the correlation between each receptor density map and FC PC1, and again compared the product of the correlations with the z-assortativity of each pair of annotations. We again find a significant relationship ($r(188) = 0.74$; $p = 6.8 \times 10^{-34}$, CI=[0.67, 0.80], two-sided). Spatial coordinates in **a** correspond to the parcel centroids of the 800-nodes Schaefer functional atlas [89]. Cortical surfaces in **a** are visualized using PySurfer [142] and represent the pial surface of the FreeSurfer [145] fsaverage template. For the scatter plots in **c** and **d**, the regression lines are shown in black with shaded bands representing the 95% confidence intervals of the regression estimates.

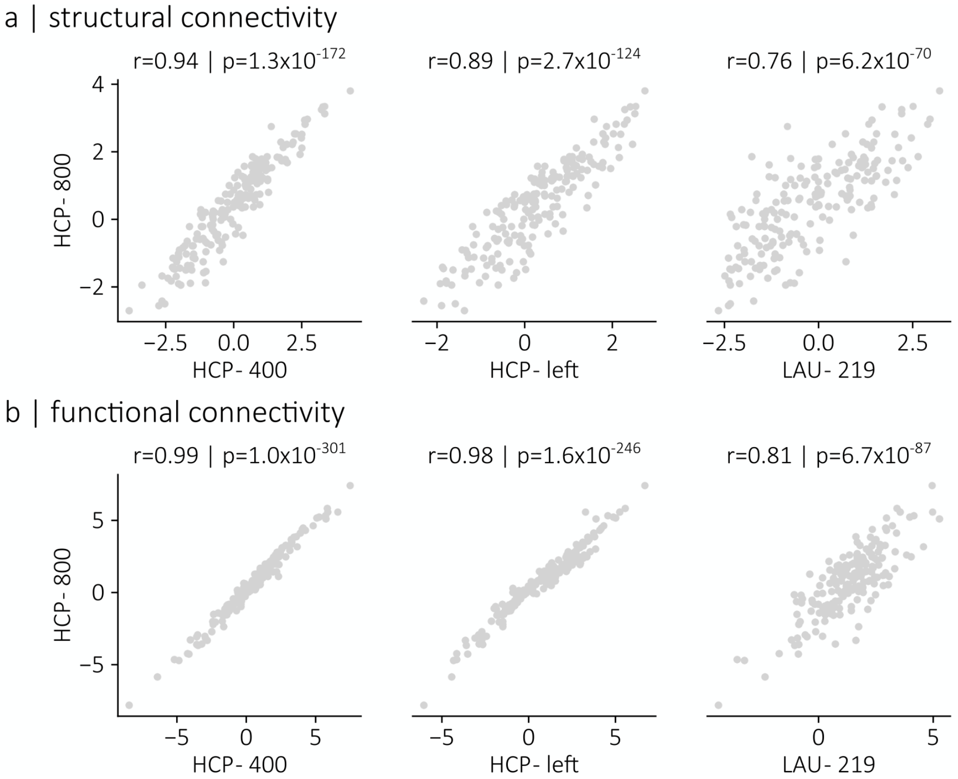

Figure S8. **Sensitivity and replication (heterophilic mixing) |** To ensure that the heterophilic relationships of receptors and transporters uncovered in the main text are replicable, we correlated the z-assortativity scores presented in the main text, which were obtained using the HCP dataset and the Schaefer 800-nodes parcellation (HCP - 800), with the z-assortativity scores obtained using an alternate parcellation namely, the 400-nodes Schaefer parcellation (HCP - 400; right). We also correlated the z-assortativity scores presented in the main text with z-assortativity scores obtained when considering only the left-hemisphere nodes of the connectome (HCP - left; middle), and when using an alternate dataset and parcellation, namely the Lausanne dataset and the 219-nodes Cammoun parcellation (LAU - 219; left). **(a)** For the structural connectome, we find significant correlations ($p < 0.05$; two-sided) for all sensitivity and replication experiments (HCP - 400: $r(359) = 0.94$, $p = 1.3 \times 10^{-172}$, CI=[0.93, 0.95]; HCP - left: $r(359) = 0.89$, $p = 2.7 \times 10^{-124}$, CI=[0.87, 0.91]; LAU - 219: $r(359) = 0.76$, $p = 6.2 \times 10^{-70}$, CI=[0.72, 0.80]) **(b)** For the functional connectome, we also find significant correlations for all sensitivity and replication experiments (HCP - 400: $r(359) = 0.99$, $p = 1.0 \times 10^{-301}$, CI=[0.986, 0.991]; HCP - left: $r(359) = 0.98$, $p = 1.6 \times 10^{-246}$, CI=[0.97, 0.98]; LAU - 219: $r(359) = 0.81$, $p = 6.7 \times 10^{-87}$, CI=[0.78, 0.85])

.

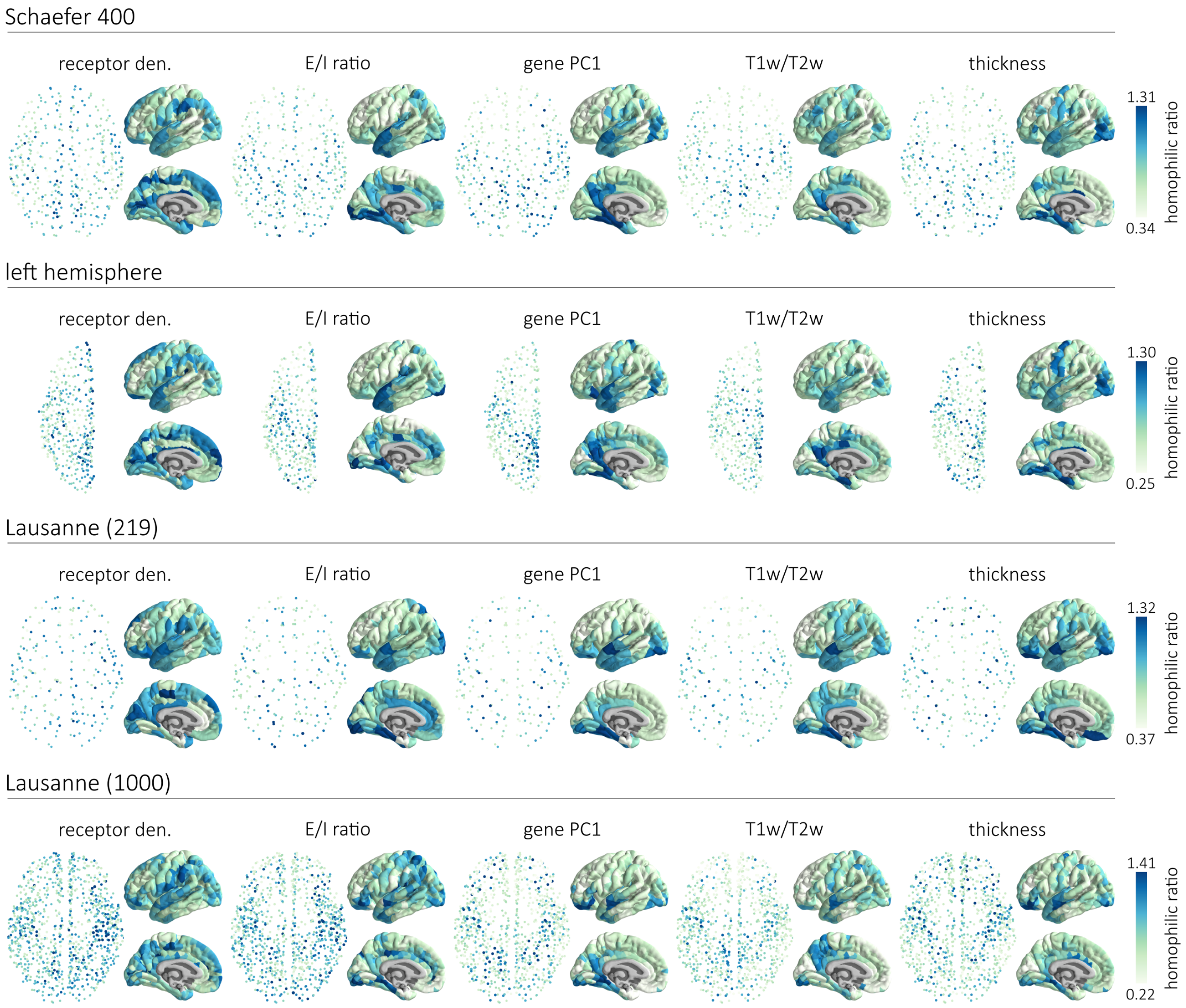

Figure S9. **Sensitivity and replication (homophilic ratios) |** To ensure that the results are not confounded by processing choices, we computed the homophilic ratios of each micro-architectural attribute in a structural connectome generated using the 400 nodes Schaefer parcellation [89], in a structural connectome of the brain's left-hemisphere, and in a structural connectome reconstructed using an independently acquired diffusion imaging dataset and parcellated into 219 and 1000 brain regions according to the Cammoun atlas [96]. The micro-architectural attributes include the density of neurotransmitter receptors, the ratio of excitatory/inhibitory receptors, the principal axis of gene transcription variation, the T1w/T2w ratio and cortical thickness. Spatial coordinates correspond to the parcel centroids of the parcellation atlases. Cortical surfaces are visualized using PySurfer [142] and represent the pial surface of the FreeSurfer [145] fsaverage template.

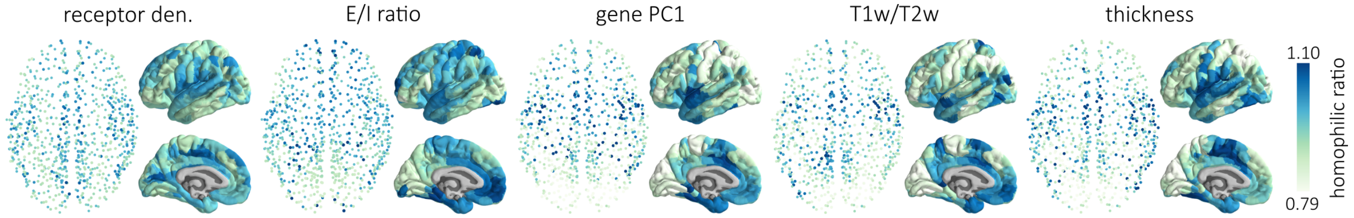

Figure S10. **Homophilic ratios in the functional connectome |** Homophilic ratios are shown for the human functional connectome. Micro-architectural attributes include the density of neurotransmitter receptors, the ratio of excitatory/inhibitory receptors, the principal axis of gene transcription variation, the T1w/T2w ratio and cortical thickness. Spatial coordinates correspond to the parcel centroids of the 800-nodes Schaefer functional atlas [89]. Cortical surfaces are visualized using PySurfer [142] and represent the pial surface of the FreeSurfer [145] fsaverage template.

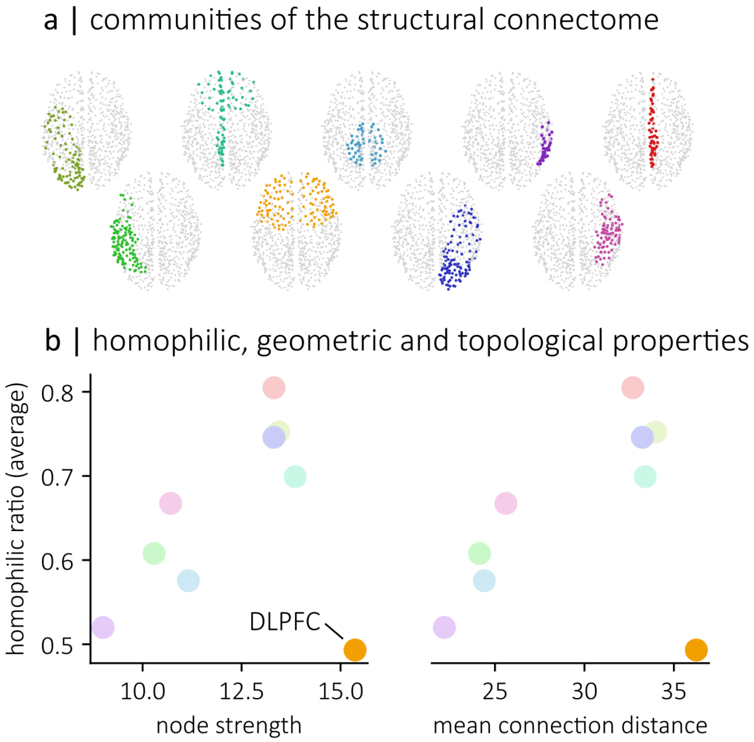

Figure S11. **Homophilic ratios in the communities of the structural connectome |** **(a)** We clustered the human structural connectome into 9 communities of highly interconnected brain regions. Spatial coordinates correspond to the parcel centroids of the 800-nodes Schaefer functional atlas [89]. **(b)** We computed the mean homophilic ratio, node strength and mean connection distance in each community and evaluated how these averaged scores relate to each other. We find positive relationships between node strength and homophilic ratio (left), as well as between mean connection distance and homophilic ratio (right). The dorsolateral prefrontal cortex (DLPFC), however, is an outlier: it has the largest mean node strengths and mean connection distances, but the smallest homophilic ratios.

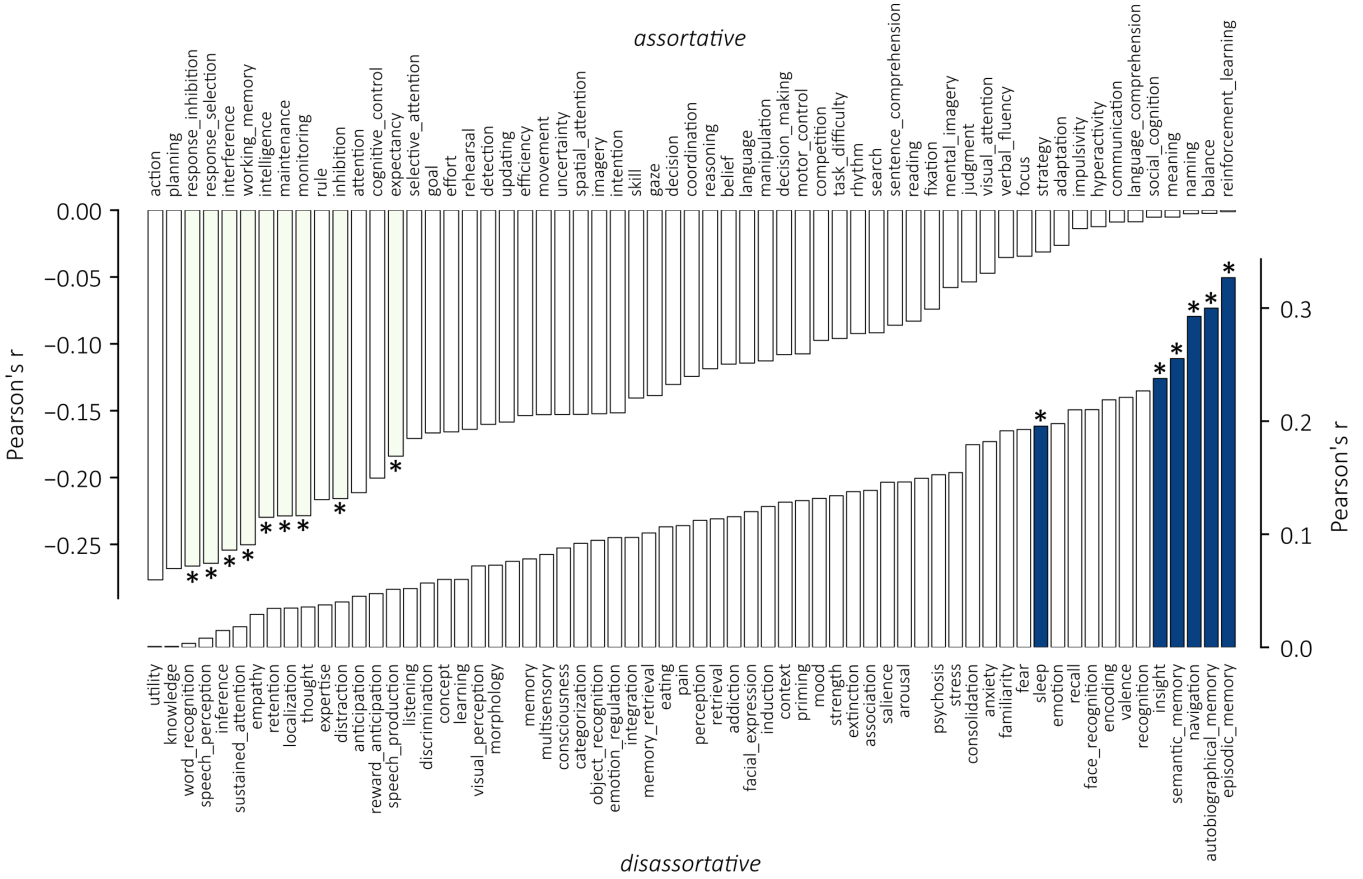

Figure S12. **Neurosynth correlations |** Correlation coefficients between the averaged homophilic ratios of micro-architectural attributes and 123 brain maps associated with cognitive and behavioural terms. Each map represents the probabilistic association between the term and individual voxel activation. We find 59 negative relationships (top) and 64 positive relationships (bottom). Asterisks and colored bars denote the significant relationships, which were evaluated against spatial autocorrelation-preserving null annotations ($p_{\text{spin}} < 0.05$, two-sided). Terms associated to brain maps with a significant negative relationship to the averaged homophilic ratios include: response inhibition ($r = -0.27$, $p_{\text{spin}} = 0.003$), response selection ($r = -0.26$, $p_{\text{spin}} = 0.008$), interference ($r = -0.25$, $p_{\text{spin}} = 0.02$), working memory ($r = -0.25$, $p_{\text{spin}} = 0.02$), intelligence ($r = -0.23$, $p_{\text{spin}} = 0.002$), maintenance ($r = -0.23$, $p_{\text{spin}} = 0.03$), monitoring ($r = -0.23$, $p_{\text{spin}} = 0.049$), inhibition ($r = -0.22$, $p_{\text{spin}} = 0.04$) and expectancy ($r = -0.18$, $p_{\text{spin}} = 0.02$). Terms associated to brain maps with a significant positive relationship to the averaged homophilic ratios include: sleep ($r = 0.20$, $p_{\text{spin}} = 0.04$), insight ($r = 0.24$, $p_{\text{spin}} = 0.002$), semantic memory ($r = 0.26$, $p_{\text{spin}} = 0.03$), navigation ($r = 0.29$, $p_{\text{spin}} = 0.03$), autobiographical memory ($r = 0.30$, $p_{\text{spin}} = 0.01$) and episodic memory ($r = 0.33$, $p_{\text{spin}} = 0.007$).
