# Supplementary material for: Assortative mixing in micro-architecturally annotated brain connectomes
Source: Nat Commun. 2023 May 18;14:2850. doi: 10.1038/s41467-023-38585-4 (PMC10195875; doi:10.1038/s41467-023-38585-4)
Supplement: Supplementary file 3 — Reporting Summary [file 41467_2023_38585_MOESM3_ESM.pdf]

nature portfolio

Corresponding author(s): Bratislav Misic

Last updated by author(s): Feb 27, 2023

Reporting Summary

Nature Portfolio wishes to improve the reproducibility of the work that we publish. This form provides structure for consistency and transparency in reporting. For further information on Nature Portfolio policies, see our Editorial Policies and the Editorial Policy Checklist.

Statistics

For all statistical analyses, confirm that the following items are present in the figure legend, table legend, main text, or Methods section.

n/a	Confirmed	
☐	☒	The exact sample size (n) for each experimental group/condition, given as a discrete number and unit of measurement
☐	☒	A statement on whether measurements were taken from distinct samples or whether the same sample was measured repeatedly
☐	☒	The statistical test(s) used AND whether they are one- or two-sided<br>*Only common tests should be described solely by name; describe more complex techniques in the Methods section.*
☐	☒	A description of all covariates tested
☐	☒	A description of any assumptions or corrections, such as tests of normality and adjustment for multiple comparisons
☐	☒	A full description of the statistical parameters including central tendency (e.g. means) or other basic estimates (e.g. regression coefficient) AND variation (e.g. standard deviation) or associated estimates of uncertainty (e.g. confidence intervals)
☐	☒	For null hypothesis testing, the test statistic (e.g. F, t, r) with confidence intervals, effect sizes, degrees of freedom and P value noted<br>*Give P values as exact values whenever suitable.*
☒	☐	For Bayesian analysis, information on the choice of priors and Markov chain Monte Carlo settings
☒	☐	For hierarchical and complex designs, identification of the appropriate level for tests and full reporting of outcomes
☐	☒	Estimates of effect sizes (e.g. Cohen's d, Pearson's r), indicating how they were calculated

Our web collection on statistics for biologists contains articles on many of the points above.

Software and code

Policy information about availability of computer code

Data collection	MRtrix3 (0.0.3) was used to reconstruct structural connectomes
Data analysis	All code used to run the analyses and generate the figures can be found at https://github.com/netneurolab/bazinet_assortativity. Analyses relied on the following open-source Python packages: BrainSMASH (0.11.0), brainspace (0.1.4), abagen (0.1.3), neuromaps (0.0.3). Data was analyzed using Python 3.9.7

For manuscripts utilizing custom algorithms or software that are central to the research but not yet described in published literature, software must be made available to editors and reviewers. We strongly encourage code deposition in a community repository (e.g. GitHub). See the Nature Portfolio guidelines for submitting code & software for further information.

Data

Policy information about availability of data

All manuscripts must include a data availability statement. This statement should provide the following information, where applicable:

- Accession codes, unique identifiers, or web links for publicly available datasets
- A description of any restrictions on data availability
- For clinical datasets or third party data, please ensure that the statement adheres to our policy

The data used to conduct the analyses is available at https://github.com/netneurolab/bazinet_assortativity. More generally, the HCP dataset is available at https://db.humanconnectome.org/data/projects/HCP_1200, the Lausanne dataset is available at https://doi.org/10.5281/zenodo.2872624, the Allen Human Brain Atlas is

available at https://human.brain-map.org, the receptor density atlas is available through neuromaps (https://github.com/netneurolab/neuromaps), the Allen Mouse Brain Connectivity Atlas is available at https://connectivity.brain-map.org, the Allen Mouse Brain Atlas is available at https://mouse.brain-map.org/static/atlas, the CoCoMac database is available at http://cocomac.g-node.org/, the macaque Neuron density data is available at https://doi.org/10.1073/pnas.1010356107, the macaque structural MRI scans are publicly available in the BALSA database (https://balsa.wustl.edu/study/show/W336) and the BigBrain data is available at https://ftp.bigbrainproject.org/.

Human research participants

Policy information about studies involving human research participants and Sex and Gender in Research.

Reporting on sex and gender	Structural and functional connectomes were group consensus networks generated from individidual connectomes of subjects of both sexes.
Population characteristics	HCP: age 28+/- 3.73 years, 55% female. Lausanne: age 28.8 +/- 9.1 years, 40% female.
Recruitment	Only data from healthy control subjects were used in the analyses.
Ethics oversight	HCP: Informed consent was obtained for all subjects. The protocol was approved by the Washington University Institutional Review Board as part of the HCP.<br>Lausanne: Informed consent was obtained for all subjects. The protocol was approved by the Ethics Committee of Clinical Research of the Faculty of Biology and Medicine, University of Lausanne, Switzerland.

Note that full information on the approval of the study protocol must also be provided in the manuscript.

Field-specific reporting

Please select the one below that is the best fit for your research. If you are not sure, read the appropriate sections before making your selection.

☒ Life sciences ☐ Behavioural & social sciences ☐ Ecological, evolutionary & environmental sciences

For a reference copy of the document with all sections, see nature.com/documents/nr-reporting-summary-flat.pdf

Life sciences study design

All studies must disclose on these points even when the disclosure is negative.

Sample size	The justification for the sample size of each dataset is provided in the original articles.<br>Human connectomes (HCP): https://doi.org/10.1016/j.neuroimage.2013.05.041<br>Human connectomes (Lausanne): https://doi.org/10.5281/zenodo.2872624<br>Macaque connectome: https://doi.org/10.1523/JNEUROSCI.0752-14.2014<br>Mouse connectome: https://doi.org/10.1038/nature13186<br>Human receptor density: https://doi.org/10.1101/2021.10.28.466336<br>Human transcriptomic: https://doi.org/10.1038/nature11405<br>Macaque morphometric: : https://doi.org/10.1523/JNEUROSCI.0493-16.2016<br>Macaque neuron density: https://doi.org/10.1073/pnas.1010356107<br>Mouse gene expression: https://doi.org/10.1038/nature05453
Data exclusions	Human connectomes (HCP): Out of the original 898 subjects of the S900 release, 82 were discarded because they had missing functional or structural scans and another 353 were discarded to remove familial relationships across subjects. In the remaining 463 subjects, 184 were monozygotic twins, so we only considered one member of each pair. 20 subjects were also removed at Quality Control, and 24 were removed because they lacked DWI images.
Replication	Analyses presented in figures 3 and 4 were replicated in eight sensitivity and replication experiments that included using a different parcellation scheme, using single-hemisphere connectomes, using an independently acquired dataset, using additional spatially-autocorrelation preserving nulls models and using a rank-based assortativity measure. Analyses presented in figure 5 were replicated in three sensitivity and replication experiments that include using a different parcellation scheme, using a single-hemisphere connectome, and using an independtly acquired dataset. Analyses presented in figure 6 were replicated in four sensitivity and replication experiments that include using different parcellation schemes, using a single-hemisphere connectome and an independently acquired dataset.
Randomization	No randomization was performed as this study does not include experimental groups.
Blinding	Blinding is not relevant to this study because it does not include experimental groups.

Reporting for specific materials, systems and methods

We require information from authors about some types of materials, experimental systems and methods used in many studies. Here, indicate whether each material, system or method listed is relevant to your study. If you are not sure if a list item applies to your research, read the appropriate section before selecting a response.

Materials & experimental systems

n/a	Involved in the study
☒	☐ Antibodies
☒	☐ Eukaryotic cell lines
☒	☐ Palaeontology and archaeology
☐	☒ Animals and other organisms
☒	☐ Clinical data
☒	☐ Dual use research of concern

Methods

n/a	Involved in the study
☒	☐ ChIP-seq
☒	☐ Flow cytometry
☐	☒ MRI-based neuroimaging

Animals and other research organisms

Policy information about studies involving animals; ARRIVE guidelines recommended for reporting animal research, and Sex and Gender in Research

Laboratory animals	For full information on laboratory animals, please refer to the original articles:<br>Macaque connectome: https://doi.org/10.1523/JNEUROSCI.0752-14.2014<br>Mouse connectome: https://doi.org/10.1038/nature13186<br>Macaque morphometric: : https://doi.org/10.1523/JNEUROSCI.0493-16.2016<br>Macaque neuron density: https://doi.org/10.1073/pnas.1010356107<br>Mouse gene expression: https://doi.org/10.1038/nature05453
Wild animals	This study did not involve wild animals
Reporting on sex	For full information on laboratory animals, please refer to the original articles:<br>Macaque connectome: https://doi.org/10.1523/JNEUROSCI.0752-14.2014<br>Mouse connectome: https://doi.org/10.1038/nature13186<br>Macaque morphometric: : https://doi.org/10.1523/JNEUROSCI.0493-16.2016<br>Macaque neuron density: https://doi.org/10.1073/pnas.1010356107<br>Mouse gene expression: https://doi.org/10.1038/nature05453
Field-collected samples	This study did not involve samples collected from the field
Ethics oversight	For full information on laboratory animals, please refer to the original articles:<br>Macaque connectome: https://doi.org/10.1523/JNEUROSCI.0752-14.2014<br>Mouse connectome: https://doi.org/10.1038/nature13186<br>Macaque morphometric: : https://doi.org/10.1523/JNEUROSCI.0493-16.2016<br>Macaque neuron density: https://doi.org/10.1073/pnas.1010356107<br>Mouse gene expression: https://doi.org/10.1038/nature05453

Note that full information on the approval of the study protocol must also be provided in the manuscript.

Magnetic resonance imaging

Experimental design

Design type	Structural MRI, resting-state fMRI and diffusion-weighted MRI
Design specifications	No trials
Behavioral performance measures	no behavioural measures

Acquisition

Imaging type(s)	Structural MRI, resting-state fMRI and diffusion-weighted MRI
Field strength	3T
Sequence & imaging parameters	HCP: the acquisition protocol included a high angular resolution imaging (HARDI) sequence and four resting state fMRI sessions. The dMRI data was acquired with a spin-echo EPI sequence (TR=5,520 ms; TE=89.5 ms; FOV=210 x 180 mm^2; voxel size=1.25 mm^3; b-value=three different shells i.e., 1,000, 2,000, and 3,000 s/mm^2; number of diffusion directions=270; and number of b0 images=18) and the resting-state fMRI data was acquired using a gradient-echo EPI sequence (TR=720 ms; TE=33.1 ms; FOV=208 x 180 mm^2; voxel size=2 mm^3; number of slices=72; and number of volumes=1,200). Additional information regarding the acquisition protocol is available at https://doi.org/10.1016/j.neuroimage.2013.05.039.

Lausanne: the data acquisition protocol included a magnetization-prepared rapid acquisition gradient echo (MPRAGE) sequence (1mm in-plane resolution, 1.2mm slice thickness), a diffusion spectrum imaging (DSI) sequence (128 diffusion-weighted volumes and a single b0 volume, maximum b-value 8,000 s/mm^2, 2.2 x 2.2 x 3.0 mm voxel size), and a gradient echo-planar imaging (EPI) sequence sensitive to blood-oxygen-level-dependent (BOLD) contrast (3.3 mm in-plane resolution and slice thickness with a 0.3-mm gap, TR 1,920 ms, resulting in 280 images per participant).

Area of acquisition: Whole-brain

Diffusion MRI ☒ Used ☐ Not used

Parameters: HCP: spin-echo EPI sequence (TR=5,520 ms; TE=89.5 ms; FOV=210 x 180 mm^2; voxel size=1.25 mm^3; b-value=three different shells i.e., 1,000, 2,000, and 3,000 s/mm^2; number of diffusion directions=270; and number of b0 images=18)

Lausanne: 128 diffusion-weighted volumes and a single b0 volume, maximum b-value 8,000 s/mm2, 2.2x2.2x3.0 mm voxel size

Preprocessing

Preprocessing software: HCP: More information regarding data preprocessing is available in: https://doi.org/10.1016/j.neuroimage.2020.117429
Lausanne: Connectome Mapper Toolkit and freesurfer

Normalization: HCP: More information regarding data preprocessing is available in: https://doi.org/10.1016/j.neuroimage.2020.117429
Lausanne: For further details about data processing, please refer to https://doi.org/10.5281/zenodo.2872624

Normalization template: HCP: More information regarding data preprocessing is available in: https://doi.org/10.1016/j.neuroimage.2020.117429
Lausanne: For further details about data processing, please refer to https://doi.org/10.5281/zenodo.2872624

Noise and artifact removal: HCP: More information regarding data preprocessing is available in: https://doi.org/10.1016/j.neuroimage.2020.117429
Lausanne: For further details about data processing, please refer to https://doi.org/10.5281/zenodo.2872624

Volume censoring: HCP: More information regarding data preprocessing is available in: https://doi.org/10.1016/j.neuroimage.2020.117429
Lausanne: For further details about data processing, please refer to https://doi.org/10.5281/zenodo.2872624

Statistical modeling & inference

Model type and settings: The assortativity of micro-architectural attributes was computed with respect to the structural and functional connectomes

Effect(s) tested: We tested whether assortativity for empirical micro-architectural attributes is greater or lower than the assortativity obtained with spatial autocorrelation-preserving null annotations

Specify type of analysis: ☒ Whole brain ☐ ROI-based ☐ Both

Statistic type for inference (See Eklund et al. 2016): NA

Correction: False Discovery Rate (Benjamini-Yekutieli procedure)

Models & analysis

n/a	Involved in the study
☐	☒ Functional and/or effective connectivity
☐	☒ Graph analysis
☐	☒ Multivariate modeling or predictive analysis

Functional and/or effective connectivity: HCP: A group-average functional connectivity matrix was constructed by concatenating the regional fMRI BOLD time series of all four resting-state sessions from all participants and computing the zero-lag Pearson correlation coefficient between each pair of brain regions.

Lausanne: A group-average functional connectivity matrix was reconstructed using the same procedure as described for the HCP dataset.

Graph analysis: Graph analysis was done on group-consensus weighted structural and functional connectomes. Connectivity measures used include: assortativity coefficient, node strength, homophilic ratio, mean connection distance and modularity.

Multivariate modeling and predictive analysis: Multilinear regression models and dominance analysis were used to explore the relationship between the different annotaitons. Principal component analysis was used to compute the main axis of variance in the functional connectivity matrix and in a gene expression x regions matrix.
